# A new (T-X$^\theta$) family of distributions: properties, discretization and estimation with applications

**DOI:** 10.1038/s41598-023-49425-2

**Published:** 2024-01-18

**Authors:** Rasha M. Mandouh, Mahmoud R. Mahmoud, Rasha E. Abdelatty

**Affiliations:** Department of Mathematical Statistics, Faculty of Graduate Studies for Statistical Research, Cairo University, Cairo, 12613 Egypt

**Keywords:** Statistics, Health care, Public health, Diseases, Cancer, Cancer models, Cancer therapy, Head and neck cancer

## Abstract

In this paper, a new class of distributions called the T-X$^\theta$ family of distributions for bounded—(0,1)—and unbounded—$(0,\infty )$—supported random variables is suggested. Some special sub-models of the proposed family are utilized. A new sub-model is selected to be studied in details. The statistical properties of the suggested family including quantile function, moments, moment generating function, order statistics and Rényi entropy are discussed. The maximum likelihood method is provided to estimate the parameters of the distribution and a Monte Carlo simulation study is used. The discretized T-X$^\theta$ family provided many sub-families and sub-models. In addition, eight real data sets are utilized to demonstrate the flexibility of the proposed continuous and discrete family’s multiple sub models.

## Introduction

Data and their distributions are the consequences of generating models. One can imagine that real generating methods are unlimited and therefore we are met with quite a few of models. Statisticians try to choose a mathematical model to fit a given data set. The more data we have, the larger our repertoire of models should be. The existence of a huge variety of data in many problems, requires numerous changes in the classical distributions producing new families of distributions. The newly found distributions may have new properties beside inheriting some advantages of the classical ones which often are special cases of the new generated distribution. This adds additional flexibility to the produced distribution, which it is hoped that it will be of benefit and provides accurate prediction. As a result, comparisons with the classical distributions were frequently in favor of the newly produced ones. Several approaches have been suggested on how to find new flexible distributions. Many alternatives have been employed; sometimes they realized there purpose through adding one or more parameters to a baseline distribution in different ways, some used transformation forms while others merged more than one distribution to obtain a new one. Reference^1^ proposed the transformed transformer’s family (T-X family). This method is derived from three functions (R, F, and W), where R and F are the cdfs of two random variables, T and X, respectively, and the W(.) function is used to connect T’s support to the range of X. The cdf and the pdf of the T-X family are given by

$$\begin{aligned} G(x)& = \int \limits _{a}^{W(F(x))} r(t) dt\quad = R(W(F(x))), \end{aligned}$$

and

$$\begin{aligned} g(x)& = r\{W(F(x))\}\frac{d}{d(x)}W(F(x)), \end{aligned}$$

where r(t) and R(t) are the pdf and cdf of the random variable T$\in [a,b]$, where $-\infty \le a<b\le \infty .$ The W(.) is monotonically and non decreasing function with rang of W = [*a*, *b*].

1. $$W(F(x))\in [a,b]$$.
2. *W*(*F*(*x*)) is differentiable and monotonically non decreasing.
3. $W(F(x))\longrightarrow$ a, as $x\longrightarrow -\infty \;$ and $\;W(F(x))\longrightarrow$ b, as $x\longrightarrow \infty .$

Several new types of distributions using various W(.) functions were introduced in the literature. Table 1 includes some members of the T-X family depends on different examples of the W(.) functions for non negative continuous random variable T.

**Table 1:** Some members of the T-X family.

W$\left( F(x)\right)$	Rang of T	The family	Author
F(x)	(0, 1)	Beta G family	^2^
		Kumaraswamy G family	^3^
$-\log \left( 1-F^{\alpha }(x)\right)$	$(0,\infty )$	Exponentiated T-X family	^4^
$\dfrac{-\log \left( 1-F(x)\right) }{1-F(x)}$	$(0,\infty )$	New Weibull-X family	^5^
$\dfrac{-\log \left( 1-F^{\alpha }(x)\right) }{1-F^{\alpha }(x)}$	$(0,\infty )$	Jamal Weibull-X family	^6^
$-\log \left( \dfrac{1-F(x)}{e^{F(x)}}\right)$	$(0,\infty )$	Weighted T-X family	^7^
$X^{\theta }F(x)$	(0, 1)	T-$X^{\theta }$ family	Proposed
	$$(0,\infty )$$		

The aim of this research is to introduce a new family of distributions called the T-X$^{\theta }$ family of distributions. The cdf, pdf, Survival and hazard functions of the new family are proposed, also some T-X$^{\theta }$ families based on different T distributions are introduced. Using different T and X functions some new models are introduced, the statistical properties of the E-X$^\theta$E distribution including Survival and hazard rate functions, the quantile function, moments, order statistics and Rényi entropy are derived. The maximum likelihood method is used for estimating the parameters and a simulation study on a variety of sample sizes for the E-X$^\theta$E distributions is investigated. The performance of several members of the discretized and continuous T-X$^\theta$ families of distributions are performed using eight different types of lifetime data sets. Finally the conclusion is provided.

## The new family

Here a new way of defining the W(x) function in the T-X family of distributions is proposed. Let T and X be non-negative random variables with cdf R(t) and F(x) and assumed the random variable T$\in (a,b)$ and X$\in (a,b)$, for $0\le a< b\le \infty$ then the cdf and the pdf of the new T-X$^{\theta }$ family of distributions can be defined as follows

1 $$\begin{aligned} G(x)& = R\{x^{\theta }F(x)\}, \end{aligned}$$

and

2 $$\begin{aligned} g(x)& = \int \limits _{0}^{x^{\theta }F(x)}r(t)dt = \left( x^{\theta }f(x)+\theta x^{\theta -1}F(x)\right) r\{{x^{\theta }F(x)}\}\nonumber \\& = \left( xf(x)+\theta F(x)\right) x^{\theta -1} r\{{x^{\theta }F(x)}\}, \end{aligned}$$

where $\theta >0$ is an additional shape parameter.

The new W(.) function is a generalization that can be defined for both of ranges (0,1) and $(0,\infty )$. One can see that the random variables T, and X should have the same range.

The Survival and hazard functions of a random variable X with T-X$^{\theta }$ family are, respectively given, by

$$\begin{aligned} s(x)& = 1-R\{x^{\theta }F(x)\}, \end{aligned}$$

and

$$\begin{aligned} h(x)& = h_{T}\{x^{\theta }F(x)\}. \end{aligned}$$

When $\theta$ = 0, and T is distributed as beta or Kumaraswamy distribution, then this family is the beta-generated or the Kumaraswamy-generated family of distributions as defined in Table 1.

Based on different T distributions Table 2 displays some members of T-X$^{\theta }$ family with the same X function.

**Table 2:** Some cdf’s of the new family based on different T distributions.

Distribution of t	cdf of t	G(x)	Range
Beta	$I_{t}(\alpha , \beta )$	$I_{x^{\theta }F(x)}(\alpha , \beta )$	(0, 1)
Kumaraswamy	$1-(1-t^{\alpha })^{\beta }$	$1-(1-(x^{\theta }F(x))^{\alpha })^{\beta }$	(0, 1)
Exponential	$1- e^{- \alpha t}$	$1- e^{-\alpha x^{\theta }F(x)}$	$(0,\infty )$
Weibull	$1- e^{- \frac{t^{\lambda }}{\beta }}$	$1- e^{- \frac{(x^{\theta }F(x))^{\lambda }}{\beta }}$	$(0,\infty )$
Lomax	$1-(1+\beta t)^{-\alpha }$	$1-(1+\beta x^{\theta }F(x))^{-\alpha }$	$(0,\infty )$
Fréchet	$e^{-\left( \frac{t}{\delta }\right) ^{-\kappa }}$	$e^{-\left( \frac{x^{\theta }F(x)}{\delta }\right) ^{-\kappa }}$	$(0,\infty )$
Burr	$1-(1+ t^{\alpha })^{-\beta }$	$1-(1+ (x^{\theta }F(x))^{\alpha })^{-\beta }$	$(0,\infty )$
Log-logistic	${1}/\left( {1+({t}/{\alpha })^{-\beta }}\right)$	${1}/\left( {1+({x^{\theta }F(x)}/{\alpha })^{-\beta }}\right)$	$(0,\infty )$
Gamma	${\gamma \left( \alpha , \beta t\right) }/{\Gamma (\alpha )}$	${\gamma \left( \alpha , \beta x^{\theta }F(x)\right) }/{\Gamma (\alpha )}$	$(0,\infty )$
Log-normal	$\dfrac{1}{2}\left[ 1+\phi \left( \dfrac{\ln t-\mu }{\sigma \sqrt{2}}\right) \right]$	$\dfrac{1}{2}\left[ 1+\phi \left( \dfrac{\ln (x^{\theta }F(x))-\mu }{\sigma \sqrt{2}}\right) \right]$	$(0,\infty )$

## Special models

This section introduces various new T-X$^\theta$ family models based on different X random variables. The Exponential-X$^\theta$Exponential distribution is studied in details and Some of its features are provided. In Table 3, several new models derived from the T-X$^{\theta }$ family of distributions are provided.

**Table 3:** Some members of the T-X$^{\theta }$ family.

Distribution of x	cdf	Range of *x*	Abbreviation
Uniform-X $^{\theta }$ beta $^{1}$	$I_{x^{\theta +1} }(\alpha , \beta )$	$0<x<1$	U-X$^{\theta }$B
Beta-X$^{\theta }$ uniform	$x^{\theta }\left( I_{x }(\alpha , \beta ) \right)$	$0<x<1$	B-X$^{\theta }$U
Beta-X$^{\theta }$ Kumaraswamy	$I_{x^{\theta } \left( 1-\left( 1-x^{\lambda }\right) ^{\gamma }\right) }(\alpha ,\beta )$	$0<x<1$	B-X$^{\theta }$Ku
Exponential-X$^{\theta }$ gamma	$1-e^{-\frac{\lambda x^{\theta } \gamma (\alpha ,\frac{x}{\beta })}{\Gamma (\alpha )}}$	$x>0$	E-X$^{\theta }$G
Gamma-X$^{\theta }$ Exponential	$\dfrac{1}{\Gamma (\alpha )}{\gamma \left( \alpha ,\frac{x^{\theta }\left( 1-e^{- x}\right) }{\beta }\right) }$	$x>0$	G-X$^{\theta }$E
Gamma-X$^{\theta }$ Lomax	$\dfrac{1}{\Gamma (\alpha )}{\gamma \left( \alpha ,\frac{\left( 1-(x+1)^{-c}\right) x^{\theta }}{\beta }\right) }$	$x>0$	G-$X^{\theta }$L
Burr-X$^{\theta }$ Exponential	$1-\left( 1+x^{\theta }\left( 1-e^{-\alpha x}\right) \right) ^{-\beta }$	$x>0$	Burr$X^{\theta }$E

The Uniform-X$^\theta$Beta distribution in Table 3 is the McDonald distribution defined by Ref.^8^ with $\theta +1 = c$

### The Exponential-X$^{\theta }$Exponential

Let the random variable T follows the Exponential distribution with parameter $\lambda = 1$, and F(x) is the cdf of the Exponential distribution with parameter $\alpha$, then the cdf and the pdf of the Exponential-$X^{\theta }$Exponential(E-$X^{\theta }$E) distribution are defined as

3 $$\begin{aligned} G(x)& = 1- e^{- x^{\theta }(1- e^{-\alpha x})}, \end{aligned}$$

and

4 $$\begin{aligned} g(x)& = \left( \alpha x^{\theta }e^{-\alpha x}+\theta x^{\theta -1}(1- e^{-\alpha x})\right) e^{-x^{\theta }(1- e^{-\alpha x})}, x>0, \end{aligned}$$

where the survival and the hazard functions are

$$\begin{aligned} s(x)& = e^{-x^{\theta }(1- e^{-\alpha x})}, \end{aligned}$$

and

$$\begin{aligned} h(x)& = \alpha x^{\theta }e^{-\alpha x}+\theta x^{\theta -1}(1- e^{-\alpha x}). \end{aligned}$$

**Figure 1:**
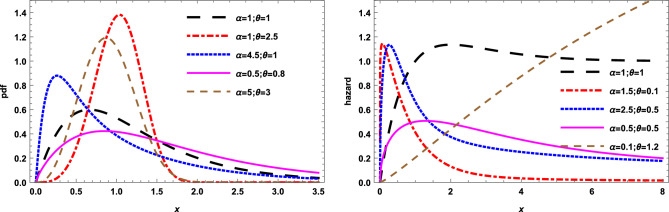
The pdf and the hazard function of the EX$^\theta$E distribution for different parameter values.

For different values of $\alpha$ and $\theta$ the pdf and the hazard function of the E-$X^{\theta }E$ distribution are plotted in Fig. 1.

### Properties of the E-X$^{\theta }$E distribution

In this subsection the E-X$^{\theta }$E distribution is studied in details. Some properties of the E-X$^{\theta }$E distribution including quantile function, moments, moment generating function, order statistics and entropy are provided.

#### The quantile function

The quantile function (qf) of the E-X$^{\theta }$E distribution can be obtained by equating the cdf (G(x)) in (3) to u, $0<u<1,$ and solve it for x, then

5 $$\begin{aligned} 1- e^{-x^{\theta }(1- e^{-\alpha x})}& = u,\nonumber \\ x^{\theta }(1- e^{-\alpha x})& = -\ln (1-u). \end{aligned}$$

There is no closed form for the quantile function and it can be obtained numerically. Butting u = 0.5, in (5), the median (M) of the E-$X^{\theta }E$ distribution can be calculated.

#### pdf expansion

Here we need to expand the pdf which will be useful in calculating some properties of the E-X$^{\theta }$E distribution such as moments and moment generating function. To obtain the pdf expansion one needs to use the following expansion

$$\begin{aligned} e^{-x^{\theta }(1- e^{-\alpha x})}& = e^{-x^{\theta }}e^{x^{\theta } e^{-\alpha x}} = \sum _{j = 0}^{\infty }\sum _{i = 0}^{\infty } \dfrac{(-1)^{j}}{j!i!} {x^{(j+i)\theta } e^{-i\alpha x}}, \end{aligned}$$

then the pdf in (4) can be expressed as

6 $$\begin{aligned} g(x)& = \sum _{i = 0}^{\infty } \sum _{j = 0}^{\infty } \frac{(-1)^j}{i! j!}\left[ \alpha x^{(i+ j+1)\theta } e^{ -( i+1)\alpha x} +\theta x^{(i+ j+1)\theta -1}e^{-\alpha i x} -\theta x^{(i+ j+1)\theta -1} e^{ -( i+1)\alpha x} \right] . \end{aligned}$$

#### The moments

If X is a random variable distributed as E-X$^{\theta }E (\alpha ,\theta )$, then the nth ordinary moment of E-X$^{\theta }E$ distribution using the pdf in (6) will be

$$\begin{aligned} E(X^{n})& = \sum _{i = 0}^{\infty } \sum _{j = 0}^{\infty } \frac{(-1)^j}{i! j!}\left[ \int _{0}^{\infty }\alpha x^{(i+ j+1) \theta +n} e^{ -( i+1)\alpha x} dx +\int _{0}^{\infty }\theta x^{( i+ j+1)\theta +n-1}e^{-\alpha i x}dx \right. \\&\quad \left. - \int _{0}^{\infty } \theta x^{( i+j+1)\theta +n-1}e^{ -(i+1)\alpha x} dx \right] ,\\ E(X^{n})& = \sum _{i = 0}^{\infty } \sum _{j = 0}^{\infty } \frac{(-1)^j}{i! j!}\left[ \frac{\alpha \Gamma ( (i+ j+1)\theta +n +1)}{(( i+1)\alpha ) ^{ (i+ j+1)\theta +n+1}}+\frac{\theta \Gamma ( (i+ j+1)\theta +n )}{(\alpha i)^{ (i+ j+1)\theta +n}} -\frac{\theta \Gamma ( (i+ j+1)\theta +n )}{( (i+1)\alpha )^{ (i+ j+1)\theta +n}} \right] . \end{aligned}$$

The central moments can be obtained from the moments by

$$\begin{aligned} E(X-\mu )^{n} = \sum _{m = 0}^{n}\left( {\begin{array}{c}n\\ m\end{array}}\right) (-1)^{m}\mu ^{n}\mu ^{'}_{n-m}. \end{aligned}$$

Therefore, the mean and the variance of the E-$x^{\theta }E$ distribution are given by

$$\begin{aligned} \mu ^{}_{}& = E(X) = \sum _{i = 0}^{\infty } \sum _{j = 0}^{\infty } \frac{(-1)^j}{i! j!}\left[ \frac{\alpha \Gamma ((i+ j+1) \theta +2)}{((i+1)\alpha )^{(i+ j+1) \theta +2}}+\frac{\theta \Gamma ((i+ j+1) \theta +1 )}{(\alpha i)^{(i+ j+1) \theta +1}} -\frac{\theta \Gamma ((i+ j+1) \theta +1 )}{((i+1)\alpha )^{(i+ j+1) \theta +1}} \right] , \end{aligned}$$

and

$$\begin{aligned} \mu ^{}_{2}& = \sigma ^{2} = E(X-\mu _{})^{2} = E(X^{2})-\mu _{}^{2}\\& = \sum _{i = 0}^{\infty } \sum _{j = 0}^{\infty } \frac{(-1)^j}{i! j!}\left[ \frac{\alpha \Gamma ((i+j+1) \theta +3)}{((i+1)\alpha )^{(i+ j+1) \theta +3}}+\frac{\theta \Gamma ((i+j+1) \theta +2 )}{(\alpha i)^{(i+ j+1) \theta +2}} -\frac{\theta \Gamma ((i+ j+1) \theta +2 )}{((i+1)\alpha )^{(i+ j+1) \theta +2}} \right] \\&\quad -\mu _{}^{2}. \end{aligned}$$

The numerical values of mean ($\mu$) and variance ($\sigma ^2$) of the E-X$^{\theta }$E distribution are listed in Table 4 for selected values of $\alpha$ and $\theta$.

**Table 4:** Mean and variance of the E-X$^{\theta }$E distribution for some values of $\alpha$ and $\theta$.

$\theta$	$\alpha$ = 0.2		$\alpha$ = 0.5		$\alpha$ = 0.8		$\alpha$ = 1		$\alpha$ = 2		$\alpha$ = 7	
	$\mu$	$\sigma ^{2}$	$\mu$	$\sigma ^{2}$	$\mu$	$\sigma ^{2}$	$\mu$	$\sigma ^{2}$	$\mu$	$\sigma ^{2}$	$\mu$	$\sigma ^{2}$
0.9	2.438	2.406	1.687	1.457	1.445	1.283	1.358	1.248	1.179	1.251	1.070	1.340
1	2.285	1.838	1.599	1.098	1.373	0.949	1.291	0.915	1.119	0.904	1.015	0.975
3	1.406	0.165	1.161	0.119	1.066	0.106	1.028	0.102	0.943	0.096	0.894	0.103
5	1.240	0.059	1.090	0.048	1.028	0.044	1.000	0.043	0.948	0.042	0.918	0.043
10	1.110	0.015	1.040	0.013	1.010	0.013	0.996	0.012	0.960	0.013	0.951	0.013

From Table 4 one can notes that; the mean and the variance of the E-X$^{\theta }E$ distribution decreases as $\theta$ or $\alpha$ increases. For fixed value of $\alpha$ or $\theta$ the mean and variance decreases as the other parameter increases.

#### The skewness and kurtosis based on moments

The measure of skewness (Sk) describes the degree of symmetry of the distribution while the kurtosis (Ku) is the peakedness of the distribution. They associated with the E-X$^{\theta }$E distribution using the moments by

$$\begin{aligned} Sk& = \dfrac{\mu ^{}_{3}}{\sigma ^{3}} = E\left[ \left( \dfrac{X-\mu ^{}_{}}{\sigma ^{}}\right) ^{3}\right] = \dfrac{E(X^{3})-3\mu E(X^{2})+3\mu ^{2} E(X^{})-\mu ^{3} }{\sigma ^{3}}, \end{aligned}$$

and

$$\begin{aligned} Ku& = \dfrac{\mu ^{}_{4}}{(\mu ^{}_{2})^{2}} = E\left[ \left( \dfrac{X-\mu ^{}_{}}{\sigma ^{}}\right) ^{4}\right] = \dfrac{E(X^{4})-4 E(X^{3})\mu +6E(X^{2})\mu ^{2}-3\mu ^{4} }{\sigma ^{4}}. \end{aligned}$$

Table 5 shows numerical values for the skewness (Sk), and kurtosis (Ku) of the E-$X^{\theta }E$ distribution for some values of $\alpha$ and $\theta$.

**Table 5:** Skewness and kurtosis of E-X$^{\theta }$E distribution for some values of $\alpha$, and $\theta$.

$\theta$	$\alpha = 0.2$		$\alpha = 0.5$		$\alpha = 0.8$		$\alpha = 1$		$\alpha = 5$		$\alpha = 7$	
	Sk	Ku	Sk	Ku	Sk	Ku	Sk	Ku	Sk	Ku	Sk	Ku
0.9	1.21	5.34	1.71	8.27	2.06	10.52	2.20	11.43	2.42	12.07	2.40	11.87
1	1.03	4.53	1.39	6.32	1.68	7.88	1.81	8.60	2.08	9.45	2.05	9.28
3	− 0.04	2.74	0.01	2.75	0.05	2.75	0.08	2.75	0.20	2.74	0.19	2.72
5	− 0.35	3.01	− 0.33	2.98	− 0.31	2.96	− 0.29	2.95	− 0.24	2.85	− 0.24	2.86
10	− 0.67	3.65	− 0.66	3.63	− 0.65	3.62	− 0.65	3.61	− 0.63	3.55	− 0.63	3.56

From the results provided in Table 5, it’s observed that the E-X$^{\theta }$E distribution covered different shapes of pdf.

For different values of $\alpha$, one can notes that; the distribution is right skew $(Sk>0)$ for $\theta <3$. For $\theta = 3$, the distribution is approximately symmetric $(Sk = 0)$, and for $\theta >3$ the distribution is left skew $(Sk<0)$.

For fixed value of $\alpha$, the kurtosis of the E-X$^{\theta }$E distribution is high peak (leptokurtic) for values of $3<\theta <5$, but the distribution is neither too high peak nor too flat topped for $3<\theta <10$ (mesokurtic).

### The moment generating function

The moment generating function of the E-X$^{\theta }$E distribution can be obtained using the expansion in (6), as follows

$$\begin{aligned} M_{X}(t) = E(e^{tX})& = \sum _{i = 0}^{\infty } \sum _{j = 0}^{\infty } \frac{(-1)^j}{i! j!} \left[ \int _{0}^{\infty }\alpha x^{(i+ j+1) \theta } e^{ -((i+1)\alpha -t)x} dx +\int _{0}^{\infty }\theta x^{(i+ j+1) \theta -1}e^{-(\alpha i-t) x}dx -\right. \nonumber \\&\left. \qquad \qquad \qquad \qquad \int _{0}^{\infty } \theta x^{(i+ j+1) \theta -1}e^{ -((i+1)\alpha -t)x} dx \right] \nonumber \\& = \sum _{i = 0}^{\infty } \sum _{j = 0}^{\infty } \frac{(-1)^j}{i! j!}\left[ \dfrac{\alpha \Gamma ((i+ j+1) \theta +1)}{((i+1)\alpha -t)^{(i+ j+1) \theta +1}} +\dfrac{\theta \Gamma ((i+ j+1) \theta )}{(\alpha i-t)^{(i+ j+1) \theta }} - \dfrac{\Gamma ((i+ j+1) \theta )}{( (i+1)\alpha -t)^{(i+ j+1) \theta }} \right] . \nonumber \end{aligned}$$

Thus we can find the nth moment by differentiating the $M_{X}(t)$ n times, and then setting t = 0 in the result; that is,

$$\begin{aligned} \dfrac{\partial }{\partial t^{n}}M_{X}(t)|_{t = 0} = E(x^{n}) = \mu '_{n}. \end{aligned}$$

### Order statistics

Let $X_{1}, \dots , X_{k}$ be k independent random variables from distribution with pdf g(x) and cdf G(x). According to Ref.^9^, the pdf of rth order statistics, is given by

7 $$\begin{aligned} g_{r:k}(x)& = \dfrac{k!}{(r-1)!(k-r)!}\left[ G(x)\right] ^{r-1}\left[ 1-G(x)\}\right] ^{k-r}g(x). \end{aligned}$$

Using the binomial expansion for te quantity

8 $$\begin{aligned} \left[ G(x)\right] ^{r-1} = \left[ 1-(1-G(x))\right] ^{r-1} = \sum _{i = 0}^{\infty }\left( {\begin{array}{c}r-1\\ i\end{array}}\right) (1-G(x))^{i}. \end{aligned}$$

Inserting (8), (3) and (4) in (7), then the pdf of the *rth* order statistic from the T-X$^{\theta }$ family of distributions is given by

$$\begin{aligned} g_{r:k}(x)& = \sum _{i = 0}^{\infty }\dfrac{k!}{(r-1-i)!(k-r)!i!}\left[ 1-R\{{x^{\theta }F(x)}\}\right] ^{i+k-r}\left( x^{\theta }f(x)+\theta x^{\theta -1}F(x)\right) r\{{x^{\theta }F(x)}\}. \end{aligned}$$

Let $X_{1}, \ldots , X_{k}$ be a random sample from the E-X$^{\theta }$E distribution. The pdf of *rth* order statistic, $X_{r:k}$, for the E-X$^{\theta }$E distribution is defined by

9 $$\begin{aligned} g_{r:k}(x) = &\sum _{l = i = j = 0}^{\infty }\omega _{i,j,l} \left[ \alpha x^{\theta +\theta i+\theta j} e^{ -(\alpha +\alpha i)x} +\theta x^{\theta +\theta i+\theta j-1}e^{-\alpha i x} -\theta x^{\theta +\theta i+\theta j-1}e^{ -(\alpha +\alpha i)x} \right] , \end{aligned}$$

where $$\omega _{i,j,l} = \dfrac{k!(-1)^j(l+k-r+1)^{i+j}}{(r-1-l)!(k-r)!l!i! j!}$$.

#### Proof

The pdf of *rth* order statistic, $X_{r:k}$, for the E-X$^{\theta }$E distribution can be written as

$$\begin{aligned} g_{r:k}(x)& = \sum _{l = 0}^{\infty }\dfrac{k!}{(r-1-l)!(k-r)!l!}\left[ e^{-x^{\theta }(1- e^{-\alpha x})}\right] ^{l+k-r}\left( \alpha x^{\theta }e^{-\alpha x}+\theta x^{\theta -1}(1- e^{-\alpha x})\right) \\&\quad \times e^{-x^{\theta }(1- e^{-\alpha x})}\\& = \sum _{l = 0}^{\infty }\dfrac{k!}{(r-1-l)!(k-r)!l!}\left[ e^{-x^{\theta }(1- e^{-\alpha x})}\right] ^{v}\left( \alpha x^{\theta }e^{-\alpha x}+\theta x^{\theta -1}(1- e^{-\alpha x})\right) , \end{aligned}$$

where $v = l+k-r+1$. Using the Exponential expansion for $e^{-vx^{\theta }+vx^{\theta } e^{-\alpha x}}$, then the Form in (9) is obtained. $\square$

### Entropy

In information theory entropy can be regarded as a measure of a system’s degree of uncertainty. It has a widely applications in economics, physics, weather science and sociology. in this section the Rényi entropy measurements is evaluated for the E-X$^{\theta }$E distribution.

### Rényi entropy

^10^ defined the Rényi entropy which considered as a generalization of the Hartley, Shannon, collision and min entropy. The Rényi entropy of a random variable X is defined by

$$\begin{aligned} I_{R}(\rho )& = \dfrac{1}{1-\rho }\log \underset{0 }{\overset{\infty }{\int }} f^{\rho }(x)dx,\rho >0,\rho \ne 1. \end{aligned}$$

The Rényi entropy of the T-$X^\theta$ family of distributions is given by

10 $$\begin{aligned} I_{R}(\rho )& = \dfrac{1}{1-\rho }\log \int _{0}^{\infty } \sum _{m = 0}^{\rho }\left( {\begin{array}{c}\rho \\ m\end{array}}\right) \theta ^m x^{\theta \rho -m}f(x)^{\rho -m}( F(x))^{m} r^{\rho }\{ x^{\theta }F(x)\} dx. \end{aligned}$$

This form is easy to be shown by applying the following binomial expansion

$$\begin{aligned} \left( x^{\theta }f(x)+\theta x^{\theta -1}F(x)\right) ^{\rho }& = \sum _{m = 0}^{\rho }\left( {\begin{array}{c}\rho \\ m\end{array}}\right) \theta ^m x^{\theta \rho -m}f(x)^{\rho -m}( F(x))^{m}. \end{aligned}$$

The Rényi entropy of of a random variable X following the E-$X^{\theta }$E distribution is

11 $$\begin{aligned} I_{R}(\rho )& = \dfrac{1}{1-\rho }\log \left[ \sum _{k = 0}^{\infty }\sum _{m = 0}^{\rho }\sum _{r = 0}^{m+k}\left( {\begin{array}{c}\rho \\ m\end{array}}\right) \left( {\begin{array}{c}m+k\\ r\end{array}}\right) \dfrac{\left( -1\right) ^{k+r}\alpha ^{(\rho -m)\Gamma \left( \theta \rho +\theta k-m+1\right) }\rho ^{k}\theta ^{m}}{k!r!\left( \alpha (\rho +r-m)\right) ^{\left( \theta \rho +\theta k-m+1\right) }} \right] . \end{aligned}$$

#### Proof

Applying result in (10), then the Rényi entropy of the E-X$^{\theta }$E can be written as

$$\begin{aligned} I_{R}(\rho )& = \dfrac{1}{1-\rho }\log \int _{0}^{\infty } \sum _{m = 0}^{\rho }\left( {\begin{array}{c}\rho \\ m\end{array}}\right) \theta ^m x^{\theta \rho -m}(\alpha e^{-\alpha x})^{\rho -m}( 1- e^{-\alpha x})^{m} e^{-\rho x^{\theta }(1- e^{-\alpha x})} dx. \end{aligned}$$

Since $e^{-\rho x^{\theta }(1- e^{-\alpha x})} = \sum _{k = 0}^{\infty }\dfrac{\left( -\rho x^{\theta }(1- e^{-\alpha x})\right) ^{k}}{k!}$ , then the Rényi entropy will be

$$\begin{aligned} I_{R}(\rho )& = \dfrac{1}{1-\rho }\log \left[ \int _{0}^{\infty } \sum _{k = 0}^{\infty }\sum _{m = 0}^{\rho }\left( {\begin{array}{c}\rho \\ m\end{array}}\right) \dfrac{\left( -1\right) ^{k}\alpha ^{(\rho -m)}\rho ^{k}\theta ^{m}}{k!} x^{\theta \rho +\theta k-m} \times e^{-\alpha (\rho -m) x}\left( 1- e^{-\alpha x}\right) ^{m+k} dx\right] . \end{aligned}$$

Since $$\left( 1- e^{-\alpha x}\right) ^{m+k} = \sum _{r = 0}^{m+k}\left( {\begin{array}{c}m+k\\ r\end{array}}\right) (-1 )^{r}e^{-r\alpha x}.$$ Then

$$\begin{aligned} I_{R}(\rho )& = \dfrac{1}{1-\rho }\log \left[ \int _{0}^{\infty } \sum _{k = 0}^{\infty }\sum _{m = 0}^{\rho }\sum _{r = 0}^{m+k}\left( {\begin{array}{c}\rho \\ m\end{array}}\right) \left( {\begin{array}{c}m+k\\ r\end{array}}\right) \dfrac{\left( -1\right) ^{k+r}\alpha ^{(\rho -m)}\rho ^{k}\theta ^{m}}{k!r!} \times x^{\theta \rho +\theta k-m}e^{-\alpha (\rho +r-m) x} dx\right] . \end{aligned}$$

Assuming the exchange between summation and integration is possible, then the last form will be

$$\begin{aligned} I_{R}(\rho )& = \dfrac{1}{1-\rho }\log \left[ \sum _{k = 0}^{\infty }\sum _{m = 0}^{\rho }\sum _{r = 0}^{m+k}\left( {\begin{array}{c}\rho \\ m\end{array}}\right) \left( {\begin{array}{c}m+k\\ r\end{array}}\right) \dfrac{\left( -1\right) ^{k+r}\alpha ^{(\rho -m)}\rho ^{k}\theta ^{m}}{k!r!} \int _{0}^{\infty } x^{\theta \rho +\theta k-m}e^{-\alpha (\rho +r-m) x} dx\right] . \end{aligned}$$

The result in Eq. (11) obtained from the last form by using the gamma function as

$$\begin{aligned} \int _{0}^{\infty } x^{\theta \rho +\theta k-m}e^{-\alpha (\rho +r-m) x} d x& = \dfrac{\Gamma \left( \theta \rho +\theta k-m+1\right) }{\left( \alpha (\rho +r-m)\right) ^{\left( \theta \rho +\theta k-m+1\right) }}. \end{aligned}$$

$$\square$$

## Parameter estimation and simulation study

In the first subsection, the maximum likelihood estimation (MLEs) of the parameters of the E-X$^{\theta }$E distribution is discussed. In the second subsection, a simulation study is obtained.

### Maximum likelihood (MLE)

Let $X_{1}, X_{2},..., X_{n}$ be a random sample from E-X$^{\theta }$E distribution. The log-likelihood function corresponding to (4) is

12 $$\begin{aligned} LL = \log L(x_{i},\alpha , \theta )& = \sum _{i = 1}^{n} \log \left( \alpha x_{i}^{\theta }e^{-\alpha x_{i}}+\theta x^{\theta -1}(1- e^{-\alpha x_{i}})\right) -\sum _{i = 1}^{n}x_{i}^{\theta }(1- e^{-\alpha x_{i}}). \end{aligned}$$

The partial derivatives of $\alpha$ and $\theta$ corresponding to (12), are given by

13 $$\begin{aligned} \dfrac{\partial }{\partial \alpha }LL& = \sum _{i = 1}^n \frac{ x_i^{\theta }e^{-\alpha x_i} - \alpha x_i^{\theta +1} e^{-\alpha x_i}+\theta x_i^{\theta } e^{-\alpha x_i} }{\alpha x_{i}^{\theta }e^{-\alpha x_{i}}+\theta x^{\theta -1}(1- e^{-\alpha x_{i}})} -\sum _{i = 1}^n x_i^{\theta +1}e^{-\alpha x_i}, \end{aligned}$$

and

14 $$\begin{aligned} \dfrac{\partial }{\partial \theta }LL& = \sum _{i = 1}^n \frac{\alpha e^{-\alpha x_i} x_i^{\theta } \log \left( x_i\right) +x_i^{\theta -1}\left( 1-e^{-\alpha x_i}\right) +\theta \left( 1-e^{-\alpha x_i}\right) x_i^{\theta -1} \log \left( x_i\right) }{\alpha x_{i}^{\theta }e^{-\alpha x_{i}}+\theta x^{\theta -1}(1- e^{-\alpha x_{i}})}\nonumber \\&\quad -\sum _{i = 1}^nx_i^{\theta } \log \left( x_i\right) \left( 1-e^{-\alpha x_i}\right) . \end{aligned}$$

Therefore, the MLE for ${\hat{\alpha }}$ and ${\hat{\theta }}$ are achieved by setting (13) and (14) to zero and then numerically solving them using a simulation technique such as Newton Rahbson.

### Interval estimation

the second derivatives of the log likelihood function for $\alpha$ and $\theta$ are

15 $$\begin{aligned} \dfrac{\partial LL}{\partial \alpha ^{2}}& = \sum _{i = 1}^n e^{-\alpha x_i} x_i^{\theta +2}+ \sum _{i = 1}^n \left[ \frac{-\theta x^2 e^{-\alpha x_i} x_i^{\theta -1}-2 e^{-\alpha x_i} x_i^{\theta +1}+\alpha e^{-\alpha x_i} x_i^{\theta +2}}{\theta \left( 1-e^{-\alpha x_i}\right) x_i^{\theta -1}+\alpha e^{-\alpha x_i} x_i^{\theta }}+\left( \theta x e^{-\alpha x_i} x_i^{\theta -1}+e^{-\alpha x_i} x_i^{\theta }-\alpha e^{-\alpha x_i} x_i^{\theta +1}\right) \times \right. \nonumber \\&\quad \left. \left( \frac{\alpha e^{-\alpha x_i} x_i^{\theta +1}}{\left( \theta \left( 1-e^{\alpha (-x)}\right) x_i^{\theta -1}+\alpha e^{-\alpha x_i} x_i^{\theta }\right) {}^2}-\frac{\theta x e^{-\alpha x} x_i^{\theta -1}}{\left( \theta \left( 1-e^{-\alpha x_i}\right) x_i^{\theta -1}+\alpha e^{-\alpha x_i} x_i^{\theta }\right) {}^2}- \frac{x_i^{\theta }e^{-\alpha x_i} }{\left( \theta x_i^{\theta -1} \left( 1-e^{-\alpha x_i}\right) +\alpha x_i^{\theta } e^{-\alpha x_i}\right) {}^2}\right) \right] , \end{aligned}$$

16 $$\begin{aligned} \dfrac{\partial ^{2}LL}{\partial \alpha \partial \theta }& = -\sum _{i = 1}^n x_i^{\theta +1} \log \left( x_i\right) e^{-\alpha x_i}+\sum _{i = 1}^n \left[ \frac{x e^{-\alpha x_i} x_i^{\theta -1}+\theta x e^{-\alpha x_i} x_i^{\theta -1} \log \left( x_i\right) +e^{-\alpha x_i} x_i^{\theta } \log \left( x_i\right) -\alpha e^{-\alpha x_i} x_i^{\theta +1} \log \left( x_i\right) }{\theta \left( 1-e^{-\alpha x_i}\right) x_i^{\theta -1}+\alpha e^{-\alpha x_i} x_i^{\theta }}+\right. \nonumber \\&\quad \left. \left( \theta x_i e^{-\alpha x_i} x_i^{\theta -1}+e^{-\alpha x_i} x_i^{\theta }-\alpha e^{-\alpha x_i} x_i^{\theta +1}\right) \left( \frac{e^{-\alpha x_i} x_i^{\theta -1}}{\left( \theta \left( 1-e^{-\alpha x_i}\right) x_i^{\theta -1}+\alpha e^{-\alpha x_i} x_i^{\theta }\right) {}^2}-\frac{x_i^{\theta -1}}{\left( \theta \left( 1-e^{-\alpha x_i}\right) x_i^{\theta -1}+\alpha e^{-\alpha x_i} x_i^{\theta }\right) {}^2}\right. \right. \nonumber \\&\quad \left. \left. +\frac{\theta e^{-\alpha x_i} x_i^{\theta -1} \log \left( x_i\right) }{\left( \theta \left( 1-e^{-\alpha x_i}\right) x_i^{\theta -1}+\alpha e^{-\alpha x_i} x_i^{\theta }\right) {}^2}- \frac{\theta x_i^{\theta -1} \log \left( x_i\right) }{\left( \theta \left( 1-e^{-\alpha x_i}\right) x_i^{\theta -1}+\alpha e^{-\alpha x_i} x_i^{\theta }\right) {}^2}-\frac{\alpha e^{-\alpha x_i} x_i^{\theta } \log \left( x_i\right) }{\left( \theta \left( 1-e^{-\alpha x_i}\right) x_i^{\theta -1}+\alpha e^{-\alpha x_i} x_i^{\theta }\right) {}^2}\right) \right] , \end{aligned}$$

and

17 $$\begin{aligned} \dfrac{\partial LL}{\partial \theta ^{2}}& = -\sum _{i = 1}^n \left( 1-e^{-\alpha x_i}\right) x_i^{\theta } \log ^2\left( x_i\right) + \sum _{i = 1}^n \left[ \frac{\theta \left( 1-e^{-\alpha x_i}\right) x_i^{\theta -1} \log ^2\left( x_i\right) +\alpha e^{-\alpha x_i} x_i^{\theta } \log ^2\left( x_i\right) +2 \left( 1-e^{-\alpha x_i}\right) x_i^{\theta -1} \log \left( x_i\right) }{\theta \left( 1-e^{-\alpha x_i}\right) x_i^{\theta -1}+\alpha e^{-\alpha x_i} x_i^{\theta }}\right. \nonumber \\&\qquad \left. +\left( \left( 1-e^{-\alpha x_i}\right) x_i^{\theta -1}+\theta \left( 1-e^{-\alpha x_i}\right) x_i^{\theta -1} \log \left( x_i\right) +\alpha e^{-\alpha x_i} x_i^{\theta } \log \left( x_i\right) \right) \times \left( \frac{e^{-\alpha x_i} x_i^{\theta -1}}{\left( \theta \left( 1-e^{-\alpha x_i}\right) x_i^{\theta -1}+\alpha e^{-\alpha x_i} x_i^{\theta }\right) {}^2}\right. \right. \nonumber \\&\qquad \left. \left. -\frac{x_i^{\theta -1}}{\left( \theta \left( 1-e^{-\alpha x_i}\right) x_i^{\theta -1}+\alpha e^{-\alpha x_i} x_i^{\theta }\right) {}^2}+\frac{\theta e^{-\alpha x_i} x_i^{\theta -1} \log \left( x_i\right) }{\left( \theta \left( 1-e^{-\alpha x_i}\right) x_i^{\theta -1}+\alpha e^{-\alpha x_i} x_i^{\theta }\right) {}^2}-\frac{\theta x_i^{\theta -1} \log \left( x_i\right) }{\left( \theta \left( 1-e^{-\alpha x_i}\right) x_i^{\theta -1}+\alpha e^{-\alpha x_i} x_i^{\theta }\right) {}^2}-\right. \right. \nonumber \\&\qquad \left. \left. \frac{\alpha e^{-\alpha x_i} x_i^{\theta } \log \left( x_i\right) }{\left( \theta \left( 1-e^{-\alpha x_i}\right) x_i^{\theta -1}+\alpha e^{-\alpha x_i} x_i^{\theta }\right) {}^2}\right) \right] . \end{aligned}$$

The hessian matrix H can be obtained as follows by using Eqs. (13), (16) and (17) .

$$\begin{aligned} H(\Theta |x) = \begin{pmatrix} \dfrac{\partial ^{2} LL}{\partial \alpha ^{2}}&{}\dfrac{\partial ^{2} LL}{\partial \alpha \partial \theta }\\ \dfrac{\partial ^{2} LL}{\partial \theta \partial \alpha }&{}\dfrac{\partial ^{2} LL}{\partial \theta ^{2}} \end{pmatrix}, \end{aligned}$$

where, ${\Theta } = (\alpha , \theta )$, $\hat{{\Theta }}\sim N_{2}({\Theta },-H(\hat{{\Theta }}|x)^{-1})$ and the information matrix $I( {\Theta }|x) = -E(H({\Theta }|x))$ , The EX$^{\theta }$E distribution’s MLEs for parameters $\alpha$ and $\theta$ only exist if the Hessian matrix is negative definite then the likelihood has a unique root. Consequently, the matrix -H($\hat{{\Theta }}$) can be used to calculate asymptotic confidence intervals for the parameters $\alpha$ and $\theta$.

### Simulation study

In this subsection a Monte Carlo simulation study is presented to demonstrate the effectiveness of the ML approach for estimating the E-X$^{\theta }E$ distribution parameters ($\alpha$ and $\theta$). The following are the steps of the simulation procedure:

1. Set the parameter values for ($\alpha$ and $\theta$) as (2,0.1), (0.1,2), (0.5,1.5), (1.2,1.5), (1,0.2), (0.3,0.2), (0.6,0.5), (0.1,0.2), (1,2), and (0.2,1).
2. Using the E-$X^{\theta }$E distribution’s quantile function, which is defined in (5), to generate a random sample of size n, where n = 20, 45, 60, 90, 120, 150, and 200.
3. Using the generated data obtained in step 2, the MLE of the parameters $\alpha$ and $\theta$ is calculated.
4. The biases and the root mean squared errors (RMSE) were determined using the provided formulas,
  $$\begin{aligned} \text {bias}(\gamma ) = \dfrac{1}{1000}\sum _{i = 1}^{1000}({\hat{\gamma }}_{i}-\gamma )\quad \text {and} \quad \text {RMSE}(\gamma ) = \sqrt{\dfrac{1}{1000}\sum _{i = 1}^{1000}({\hat{\gamma }}_{i}-\gamma )^{2}}. \end{aligned}$$

The biases, root mean squared errors and variances of ${\hat{\alpha }}$ and ${\hat{\theta }}$ are reported in Table 6. In general, as predicted, the results in this table showed a decrease in the values of the biases RMSE and variance as sample size increases, indicating that the MLE is a reliable approach for estimating the E-X$^{\theta }$E parameters as it is unbiased, the variance is minimum and it realizes the consistence.

**Table 6:** Summary of MLE’s results simulation of the E-X$^{\theta }$E for some values of $\alpha$ and $\theta$.

Value		n	$\mid$Bias$\mid$		RMSE		Variance		Value		n	$\mid$bias$\mid$		RMSE		Variance	
$\alpha$	$\theta$		${\hat{\alpha }}$	${\hat{\theta }}$	$\alpha$	$\theta$	$\alpha$	$\theta$	$\alpha$	$\theta$		${\hat{\alpha }}$	${\hat{\theta }}$	$\alpha$	$\theta$	$\alpha$	$\theta$
2	0.1	30	0.193	0.009	0.884	0.03	0.744	8.1 $\times 10^{-4}$	0.3	0.2	30	0.025	0.020	0.134	0.053	0.015	2.5 $\times 10^{-3}$
		45	0.111	0.004	0.617	0.019	0.368	3.4 $\times 10^{-4}$			45	0.013	0.013	0.093	0.040	8.5 $\times 10^{-3}$	1.5 $\times 10^{-3}$
		60	0.081	0.004	0.516	0.015	0.261	2.1 $\times 10^{-4}$			60	0.009	0.009	0.076	0.034	5.5 $\times 10^{-3}$	1.1 $\times 10^{-3}$
		90	0.038	0.003	0.403	0.012	0.161	1.36 $\times 10^{-4}$			90	0.007	0.005	0.06	0.024	3.5 $\times 10^{-3}$	5.7 $\times 10^{-4}$
		120	0.048	0.002	0.359	0.01	0.127	9.9 $\times 10^{-5}$			120	0.004	0.004	0.051	0.021	2.6 $\times 10^{-3}$	4.2 $\times 10^{-4}$
		150	0.04	0.001	0.310	0.009	0.094	7.5 $\times 10^{-5}$			150	0.006	0.003	0.046	0.018	2.1 $\times 10^{-3}$	3.2 $\times 10^{-4}$
		200	0.009	0.001	0.265	0.008	0.070	5.5 $\times 10^{-5}$			200	0.002	0.002	0.038	0.016	1.5 $\times 10^{-4}$	2.5 $\times 10^{-4}$
0.1	2	30	0.0004	0.135	0.048	0.456	2.3 $\times 10^{-3}$	0.19	0.6	0.5	30	0.077	0.055	0.515	0.124	0.259	0.013
		45	0.0002	0.073	0.037	0.353	1.4 $\times 10^{-3}$	0.112			45	0.038	0.032	0.25	0.093	0.062	7.8 $\times 10^{-3}$
		60	0.0004	0.050	0.032	0.300	1.0 $\times 10^{-3}$	0.085			60	0.02	0.021	0.17	0.070	0.031	5.2 $\times 10^{-3}$
		90	0.0006	0.044	0.025	0.230	6.4 $\times 10^{-4}$	0.051			90	0.18	0.011	0.145	0.057	0.021	3.1 $\times 10^{-3}$
		120	0.0002	0.033	0.022	0.190	4.8 $\times 10^{-4}$	0.035			120	0.009	0.009	0.112	0.049	0.012	2.3 $\times 10^{-3}$
		150	0.0007	0.017	0.019	0.16	3.8 $\times 10^{-4}$	0.027			150	0.014	0.007	0.102	0.043	0.010	1.7 $\times 10^{-3}$
		200	0.0006	0.024	0.017	0.15	2.8 $\times 10^{-4}$	0.022			200	0.0037	0.006	0.088	0.037	7.7 $\times 10^{-3}$	1.4 $\times 10^{-4}$
0.5	1.5	30	0.026	0.102	0.362	0.311	0.13	0.087	0.1	0.2	30	0.003	0.022	0.034	0.057	1.2 $\times 10^{-3}$	2.8 $\times 10^{-3}$
		45	0.0047	0.077	0.161	0.231	0.026	0.047			45	0.002	0.012	0.026	0.04	6.7 $\times 10^{-4}$	1.5 $\times 10^{-3}$
		60	0.0087	0.079	0.154	0.243	0.024	0.035			60	0.002	0.008	0.023	0.031	5.4 $\times 10^{-4}$	9.1 $\times 10^{-4}$
		90	0.0053	0.033	0.104	0.153	0.011	0.022			90	0.0002	0.007	0.019	0.026	3.6 $\times 10^{-4}$	6.5 $\times 10^{-4}$
		120	0.0055	0.022	0.087	0.129	7.5 $\times 10^{-3}$	0.016			120	0.0001	0.004	0.016	0.02	2.5 $\times 10^{-4}$	3.9 $\times 10^{-4}$
		150	0.0002	0.018	0.078	0.112	6 $\times 10^{-3}$	0.012			150	0.00007	0.004	0.014	0.019	1.9 $\times 10^{-4}$	3.5 $\times 10^{-4}$
		200	0.0005	0.010	0.068	0.101	4.6 $\times 10^{-3}$	0.010			200	0.00003	0.002	0.012	0.015	1.3 $\times 10^{-4}$	2.3 $\times 10^{-4}$
1.2	1.5	30	0.491	0.125	2.054	0.303	3.97	0.077	1	2	30	0.153	0.131	0787	0.382	0.596	0.129
		45	0.189	0.074	0.895	0.238	0.765	0.051			45	0.089	0.103	0.524	0.3	0.265	0.079
		60	0.234	0.062	0.592	0.191	0.339	0.032			60	0.047	0.068	0.366	0.233	0.132	0.050
		90	0.087	0.037	0.407	0.149	0.158	0.021			90	0.036	0.043	0.262	0.187	0.067	0.033
		120	0.056	0.028	0.33	0.128	0.107	0.016			120	0.03	0.028	0.213	0.159	0.045	0.024
		150	0.035	0.024	0.265	0.110	0.069	0.012			150	0.018	0.026	0.196	0.145	0.038	0.020
		200	0.014	0.017	0.208	0.091	0.043	7.9 $\times 10^{-3}$			200	0.011	0.020	0.168	0.125	0.028	0.015
1	0.2	30	0.159	0.022	0.573	0.061	0.303	3.2$\times 10^{-3}$	0.2	1	30	0.001	0.084	0.076	0.235	5.7 $\times 10^{-3}$	0.048
		45	0.081	0.011	0.506	0.04	0.249	1.5 $\times 10^{-3}$			45	0.003	0.058	0.062	0.177	3.8 $\times 10^{-3}$	0.028
		60	0.046	0.009	0.274	0.032	0.073	9.5 $\times 10^{-4}$			60	0.004	0.048	0.05	0.149	2.5 $\times 10^{-3}$	0.020
		90	0.033	0.004	0.218	0.025	0.046	5.9 $\times 10^{-4}$			90	0.003	0.033	0.042	0.124	1.7 $\times 10^{-3}$	0.014
		120	0.034	0.006	0.196	0.021	0.037	4.2 $\times 10^{-4}$			120	0.0001	0.021	0.037	0.099	1.4 $\times 10^{-3}$	9.2$\times 10^{-3}$
		150	0.018	0.003	0.171	0.018	0.029	3.3 $\times 10^{-4}$			150	0.0021	0.019	0.031	0.088	9.8$\times 10^{-4}$	7.3$\times 10^{-4}$
		200	0.016	0.001	0.145	0.016	0.021	2.4 $\times 10^{-4}$			200	0.0016	0.009	0.03	0.073	8.8 $\times 10^{-4}$	5.3 $\times 10^{-4}$

## Discrete T-X$^{\theta }$ family

Statistical literature contains many techniques that can be used to discretize the continuous family of distributions. One of these techniques is the one that depends on the survival function. Following^11^, the survival function for a discrete life time distribution is defined as $S(x) = P(X\ge x), x = 1,2,...$ and $S(0) = 1$, then the probability mass function (pmf) is:

18 $$\begin{aligned} P(X = x)& = P(x\le X\le x+1)\nonumber \\& = S(x)-S(x+1), x = 0,1,2,\ldots . \end{aligned}$$

The new family is generated by discretizing the continuous cdf function in (1) using the form in (18). The pmf of the T-X$^{\theta }$ family is given by

19 $$\begin{aligned} p(x)& = R\{(x+1)^{\theta }F(x+1)\}-R\{x^{\theta }F(x)\},x = 0,1,2,\ldots . \end{aligned}$$

Based on this pmf, with different T distributions, Table 7 contains some new discrete sub-families of the discrete T-X$^{\theta }$ family.

**Table 7:** Some pmfs of the new family based on different T distributions.

Distribution of t	pmf	Sub family
Exponential	$e^{- \alpha {x^{\theta }F(x)}}- e^{- \alpha {(x+1)^{\theta }F(x+1)}}$	DE-$X^{\theta }$
Weibull	$e^{- \left( \frac{x^{\theta }F(x)}{\beta }\right) ^{\lambda }}- e^{- \left( \frac{(x+1)^{\theta }F(x+1)}{\beta }\right) ^{\lambda }}$	DW-X$^{\theta }$
Rayleigh	$e^{- \left( \frac{x^{\theta }F(x)}{\beta }\right) ^{2}}- e^{- \left( \frac{(x+1)^{\theta }F(x+1)}{\beta }\right) ^{2}}$	DR-X$^{\theta }$
Fréchet	$e^{-\left( \frac{(x+1)^{\theta }F(x+1)}{\delta }\right) ^{-\kappa }}-e^{-\left( \frac{x^{\theta }F(x)}{\delta }\right) ^{-\kappa }}$	DFr-X$^{\theta }$
Lomax	$(1+\beta x^{\theta }F(x))^{-\alpha }-(1+\beta (x+1)^{\theta }F(x+1))^{-\alpha }$	DL-X$^{\theta }$
Burr	$(1+ (x^{\theta }F(x))^{\alpha })^{-\beta } -(1+ ((x+1)^{\theta }F(x+1))^{\alpha })^{-\beta }$	DB-X$^{\theta }$
Log-logistic	${1}/\left( {1+({(x+1)^{\theta }F(x+1)}/{\alpha })^{-\beta }}\right) -{1}/\left( {1+({x^{\theta }F(x)}/{\alpha })^{-\beta }}\right)$	DLL-X$^{\theta }$
Gamma	${\gamma \left( \alpha , \beta (x+1)^{\theta }F(x+1)\right) }/{\Gamma (\alpha )}-{\gamma \left( \alpha , \beta x^{\theta }F(x)\right) }/{\Gamma (\alpha )}$	DG-X$^{\theta }$
Log-normal	$\dfrac{1}{2}\left[ \Phi \left( \dfrac{\ln ((x+1)^{\theta }F(x+1))-\mu }{\sigma \sqrt{2}}\right) -\Phi \left( \dfrac{\ln (x^{\theta }F(x))-\mu }{\sigma \sqrt{2}}\right) \right]$	DLN-X$^{\theta }$

From Table 7 for the DW-X$^{\theta }$ family, we notes that;

- When the shape parameter $\lambda = 1,$ the DW-X$^{\theta }$ family reduces to the discrete Exponential family of distributions (DE-X$^{\theta }$) with parameters $1/\beta$ and $\theta$.
- When $\lambda = 2,$ the DW-X$^{\theta }$ family reduces to the discrete Rayleigh family of distributions (DR-X$^{\theta }$) with parameters $\beta ^2$ and $2\theta$.
- The DW-X$^{\theta }$ family can be considered as The DE-X$^{\theta }$ family with exponentiation F(x), ie $\theta ^{\lambda } = \theta ^{\star },$ ${\beta }^{\lambda } = {\beta }^{\star }$and $\lambda$ is the exponentiation parameter.

### The discrete exponetial-X$^{\theta }$, DE-X$^{\theta }$ family

The cumulative distribution function (cdf), survival function and probability mass function (pmf) of the DE-X$^{\theta }$ family of distributions are

20 $$\begin{aligned} G(x)& = 1- e^{- \alpha {x^{\theta }F(x)}}, x = 0,1,2,\ldots , \end{aligned}$$

21 $$\begin{aligned} p(x)& = e^{- \alpha {x^{\theta }F(x)}}- e^{- \alpha {(x+1)^{\theta }F(x+1)}} , \alpha , \theta >0. \end{aligned}$$

The survival and the hazard rate functions of the DE-X$^{\theta }$ family of distributions are

$$\begin{aligned} S(x)& = p(X\ge x) = e^{- \alpha {x^{\theta }F(x)}},\\ h(x)& = P(X = x|X\ge x) = 1-e^{-\alpha (x+1)^{\theta }F(x+1)-(x)^{\theta }F(x)}. \end{aligned}$$

With different X random variables, many distributions can be generated as members of the DE-X$^{\theta }$ family as shown in Table 8.

**Table 8:** Some pmfs of the DE-X$^{\theta }$ family based on different X distributions.

Distribution of x	pmf	Abbreviation
Exponential	$e^{-\alpha x^{\theta } \left[ 1-e^{- \beta x}\right] } - e^{-\alpha (x+1)^{\theta }\left[ 1-e^{- \beta (x+1)}\right] }$	DEE
Weibull	$e^{-\alpha x^{\theta } \left[ 1-e^{- \left( \frac{x}{\beta }\right) ^{\eta }}\right] } - e^{-\alpha (x+1)^{\theta }\left[ 1-e^{- \left( \frac{x+1}{\beta }\right) ^{\eta }}\right] }$	DEW
Fréchet	$e^{-\alpha x^{\theta } \left[ e^{- \left( \frac{x}{\beta }\right) ^{-\eta }}\right] } - e^{-\alpha (x+1)^{\theta }\left[ e^{- \left( \frac{x+1}{\beta }\right) ^{-\eta }}\right] }$	DEFr
Gamma	$e^{-\alpha x^{\theta } \left[ \frac{\Gamma {(\eta ,\beta x)}}{\Gamma {(\eta )}}\right] } - e^{-\alpha (x+1)^{\theta }\left[ \frac{\Gamma {(\eta ,\beta (x+1))}}{\Gamma {(\eta )}}\right] }$	DEG
Lomax	$e^{-\alpha x^{\theta } \left[ 1-\left( 1+ \beta x\right) ^{-\eta }\right] } - e^{-\alpha (x+1)^{\theta }\left[ 1-(1+ \beta (x+1))^{-\eta }\right] }$	DEL
Burr	$e^{-\alpha x^{\theta } \left[ 1-\left( 1+ x^{\beta }\right) ^{-\eta }\right] } - e^{-\alpha (x+1)^{\theta }\left[ 1-(1+ (x+1)^{\beta })^{-\eta }\right] }$	DEB

### The DE-Exponential$^{\varvec{\theta }}$(DEE) distribution

The DEE distribution is derived here as an example from Table 8. Substituting F(x) in (20) and (21) by the Exponential distribution with parameter $\beta$, then The pmf of the DEE distribution with three parameters are given by;

22 $$\begin{aligned} G(x)& = 1- e^{- \alpha {x^{\theta }(1-e^{-\beta x})}}, x = 0,1,2,\ldots ,\nonumber \\ p(x)& = e^{- \alpha {x^{\theta }(1-e^{-\beta x})}}- e^{- \alpha {(x+1)^{\theta }(1-e^{-\beta (x+1)})}} , \alpha , \theta , \beta >0, \end{aligned}$$

while the survival and the hazard functions are given by

$$\begin{aligned} S(x)& = p(X\ge x) = e^{- \alpha {x^{\theta }(1-e^{-\beta x})}}\nonumber \\ h(x)& = 1-e^{-\alpha (x+1)^{\theta }(1-e^{-\beta (x+1)})-(x)^{\theta }(1-e^{-\beta x})}. \end{aligned}$$

**Figure 2:**
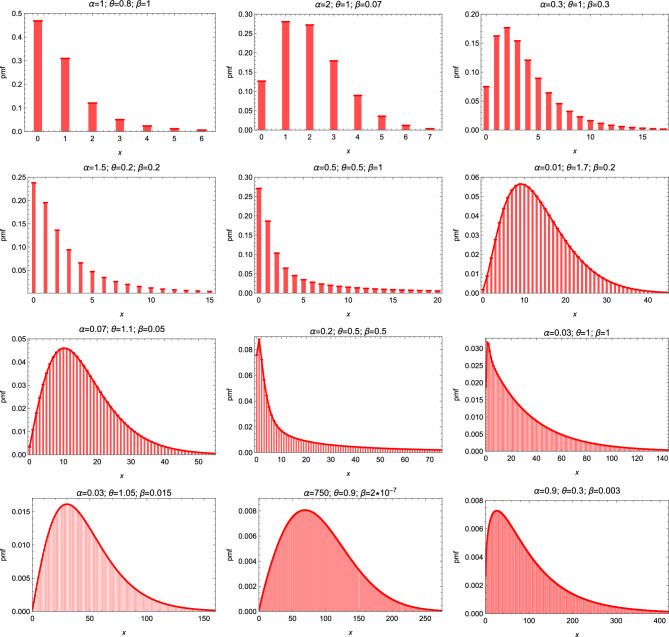
The pmf of the DEE distribution for different parameter values.

**Figure 3:**
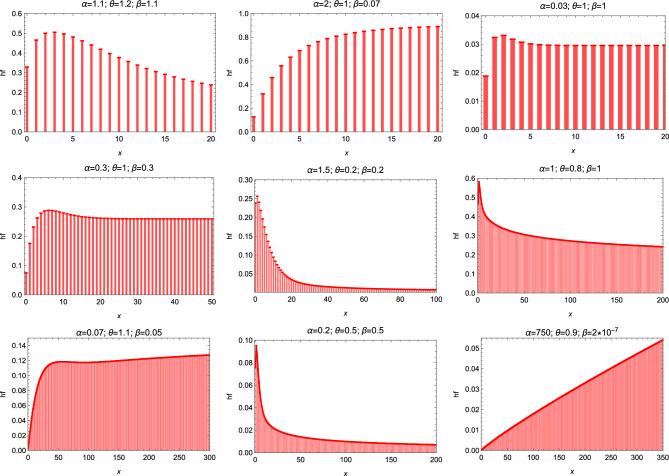
The hazard function of the DEE distribution for different parameter values.

Figure 2 displays some possible pmf shapes of the DEE distribution. The hazard rate function may has an increasing or decreasing shape as shown in Fig. 3.

## Applications to real data

The applications in this section are derived into two subsections, the first subsection contains applications of the continuous distributions displayed in Table 3. While, the second subsection deals with count data that matched the discrete distributions presented in Table 8.

### Using continuous data

In this subsection multiple models of the T-X$^\theta$ family are fitted to four different data sets. These examples are provided to demonstrate the flexibility of the new family members when compared against a variety of distributions. The estimation of each model parameters is obtained using the maximum likelihood method. To compare the distributions, three criteria are calculated, including: the Akaike information criterion (AIC), the Bayesian information criterion (BIC) and the corrected Akaike information criterion (AICc).

$$\begin{array}{*{20}l} \text {AIC} = 2k -2L(\varvec{\theta }; x), \\ \text {BIC} = \ln (nk) -2L(\theta ; x), \\ \text {AICc} = \text {AIC}+\frac{2 k(k+1) }{n-k-1}, \end{array}$$

where $L(\theta ; x)$ denotes the log likelihood for the model, k is the number of parameters and n is the sample size. In general the model that best fits the data is the one with the highest log L and p values and the lowest AIC, AICc, BIC, and Ks values. The Mathematica package was used to assess all of the required computations and figures. Table 9 shows the distributions that have been fitted to the data for comparison considerations.

**Table 9:** Competitive distributions.

Model	Abbreviation	cdf	By
Lomax–Gumbel{Fréchet}	L-G{F}	$1-{\left( 1+\gamma e^x\right) ^{-\alpha }}$	^12^
Compound inverse Rayleigh	CIR	$\left( 1+\frac{ x^{-2}}{\alpha }\right) ^{-\beta }$	^13^
Power inverse Lomax	PIL	$\left( 1+\frac{\beta }{x^{\theta }}\right) ^{-\alpha }$	^14^
New weighted Lomax	NWL	$1-\left( \frac{\beta \left( \frac{e^x}{\beta }+1\right) }{\beta +1}\right) ^{-\alpha }$	^15^

### Data set I

The data set provides the wait times (in min) before service for 100 Bank clients, which were evaluated and assessed by^16^ for fitting the Lindley distribution. The data are: 0.8, 0.8, 1.3, 1.5, 1.8, 1.9, 1.9, 2.1, 2.6, 2.7, 2.9, 3.1,3.2, 3.3, 3.5, 3.6, 4.0, 4.1, 4.2, 4.2, 4.3, 4.3, 4.4, 4.4, 4.6, 4.7, 4.7, 4.8, 4.9 ,4.9, 5.0, 5.3, 5.5, 5.7, 5.7, 6.1, 6.2, 6.2, 6.2, 6.3, 6.7 ,6.9 ,7.1, 7.1, 7.1, 7.1, 7.4, 7.6, 7.7, 8.0, 8.2, 8.6, 8.6, 8.6, 8.8, 8.8, 8.9, 8.9, 9.5, 9.6,9.7, 9.8, 10.7, 10.9, 11, 11, 11.1, 11.2, 11.2, 11.5, 11.9, 12.4,12.5, 12.9, 13, 13.1, 13.3, 13.6, 13.7, 13.9, 14.1, 15.4, 15.4, 17.3,17.3, 18.1, 18.2, 18.4, 18.9, 19, 19.9, 20.6, 21.3, 21.4, 21.9, 23,27, 31.6, 33.1, 38.5.

**Figure 4:**
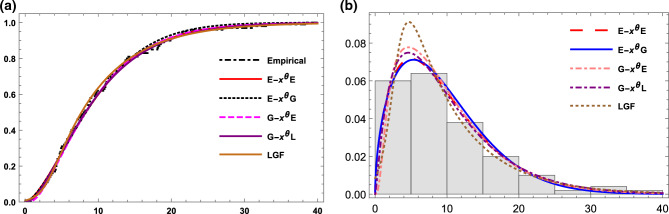
(**a**) The empirical and the estimated cdf for data set I. (**b**) The histogram and estimated pdf for data set I.

The estimated parameter values and the goodness of fit measures for this data are presented in Table 10. According to the results in this table, the G-X$^\theta$L, E-X$^\theta$E, G-X$^\theta$E, E-X$^\theta$G, and L-G{F} distributions are fitted to this data. The G-X$^\theta$L distribution is the best option among other competitive models, as shown by the findings in Table 10, as it has the greatest p-value, and the smallest other goodness of fit statistics. These findings are also supported by Fig. 4 which represents the empirical cdf and the observed density (histogram) for data set I together with the competitive models.

**Table 10:** Goodness-of fit statistics for data set I.

Model	Estimates	-${\hat{ll}}$	AIC	BIC	AICc	K-S	p-value
G-X$^{\theta }$L$(\alpha , \theta , \beta ,c)$	(1.255, 1, 4.15, 0.29)	317.16	640.3	640.6	640.0	0.041	0.994
E-X$^{\theta }$E$(\alpha , \theta )$	(0.026, 0.602)	317.89	639.7	639.9	641.1	0.043	0.987
G-X$^{\theta }$E$(\alpha , \theta , \beta )$	(2.03, 1.015, 5)	317.60	641.2	641.4	640.9	0.048	0.969
E-X$^{\theta }$G$(\alpha , \theta , \beta )$	(0.356, 1.54, 1.324)	319.36	644.7	644.9	644.4	0.052	0.933
L-G{F}($\alpha , \gamma$)	(0.1392, 0.0657)	318.55	642.2	642.3	643.5	0.051	0.949

### Data set II

The data set comes from Ref.^17^ and represents the time between failures for repairable components. The data are provided as shown below:

- 1.43, 0.11, 0.71, 0.77, 2.63, 1.49, 3.46, 2.46, 0.59, 0.74, 1.23, 0.94, 4.36, 0.40, 1.74, 4.73, 2.23, 0.45, 0.70, 1.06, 1.46, 0.30, 1.82, 2.37, 0.63, 1.23, 1.24, 1.97, 1.86, 1.17.

**Figure 5:**
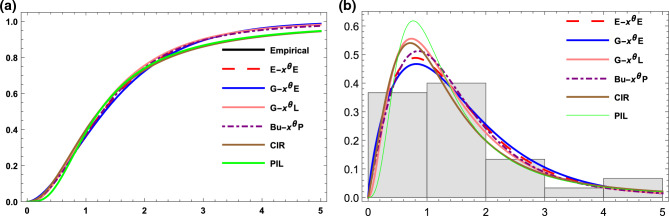
(**a**) The empirical and the estimated cdf for data set II. (**b**) The histogram and estimated pdf for data set II.

The parameter estimates and the goodness-of-fit statistics for E-X$^{\theta }$E, G-X$^{\theta }$L,G-X$^{\theta }$E, Bu-X$^{\theta }$E, CIR and PIL distributions are listed in Table 11. All competitive distributions are fitted and perform well when examining these data with a p-value greater than 0.05. However, the optimal model to acquire the best assessment of the data is the E-X$^{\theta }$E model, which has the smallest values of -ll, AIC, BIC, AICc and k-s statistics, as well as the highest p-value of all the examined models. Figure 5 supports the results in Table 11.

**Table 11:** Goodness-of fit statistics for data set II.

Model	Estimates	-${\hat{ll}}$	AIC	BIC	AICc	K-S	p-value
E-X$^{\theta }$E$(\alpha , \theta )$	(0.621, 0.928)	39.6	83.3	83.4	84.6	0.072	0.995
G-X$^{\theta }$L$(\alpha , \theta , \beta , c)$	(1.84, 0.665, 0.45, 1.155)	39.96	87.9	88.3	85.9	0.077	0.988
G-X$^{\theta }$E$(\alpha , \theta , \beta )$	(0.778, 1.33, 2.205)	39.6	85.2	85.5	84.9	0.078	0.986
Bu-X$^{\theta }$E$(\alpha , \theta , \beta )$	(0.226, 1.004, 2.485)	39.87	85.7	86	85.6	0.083	0.975
CIR($\alpha , \beta$)	(0.718, 1.05)	40.68	85.4	85.8	88.2	0.078	0.985
PIL($\alpha , \theta , \beta$)	(0.615, 4, 2.8)	39.89	85.7	86.8	89.9	0.080	0.983

### Data set III

The data set was acquired from Ref.^18^and consists of 30 successive values of March precipitation (in inches) in Minneapolis/St Paul. The data is as follows:

- 0.77, 1.74, 0.81, 1.2, 1.95, 1.2, 0.47, 1.43, 3.37, 2.2, 3, 3.09, 1.51, 2.1, 0.52, 1.62, 1.31, 0.32, 0.59, 0.81, 2.81, 1.87, 1.18, 1.35, 4.75, 2.48, 0.96, 1.89, 0.9, 2.05.

**Figure 6:**
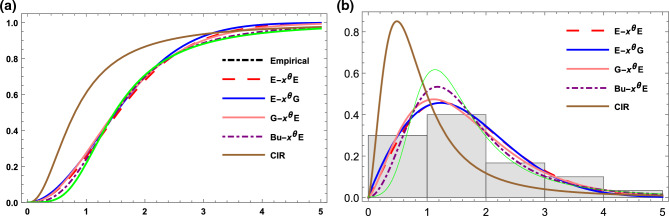
(**a**) The empirical and the estimated cdf for data set III. (**b**) The histogram and estimated pdf for data set III.

Figure 6 depicts the empirical cdf and observed density (histogram) for Data set III, compared with the cdf’s and pdf’s of E-X$^{\theta }$E, E-X$^{\theta }$G, G-X$^{\theta }$E, Bu-X$^{\theta }$E and CIR distributions. Table 12 displays the calculated parameters as well as the goodness-of-fit values. As seen in Table 12 and Fig. 6, the G-X$\theta$E distribution was chosen as the best model for this data because it had the lowest goodness-of-fit statistics and the greatest p-value, which = 1, of all the competitive distributions.

**Table 12:** Parameter estimate and goodness-of fit statistics data set III.

Model	Estimates	-${\hat{ll}}$	AIC	BIC	AICc	K-S	p-value
E-X$^{\theta }$E$(\alpha , \theta )$	(0.348, 1.185)	38.37	80.73	80.83	81.18	0.063	0.999
E-X$^{\theta }$G$(\alpha , \theta ,{\beta })$	(0.857, 1.905, 0.87)	38.86	83.7	82.2	84.6	0.065	0.999
G-X$^{\theta }$E$({\alpha },{\theta },{\beta })$	(1, 1.425, 1.96)	38.17	82.35	80.85	83.2	0.061	1
BU-X$^{\theta }$E$(\alpha , \beta \theta )$	(0.254, 1.36, 1.83)	38.82	83.62	82.14	84.56	0.083	0.973
CIR(${\alpha },{\beta }$)	(1.198, 1.97)	39.96	83.93	86.72	84.37	0.097	0.914

### Data set IV

The data shows the remission times (in months) of 128 bladder cancer patients. Reference^15^ have made use of this data. The following are the data set values:

- 0.08, 2.09, 3.48, 4.87, 6.94, 8.66, 13.11, 23.63, 0.20, 2.23, 3.52, 4.98, 6.97, 9.02, 13.29, 0.40, 2.26, 3.57, 5.06, 7.09, 9.22, 13.80, 25.74, 0.50, 2.46, 3.64, 5.09, 7.26, 9.47, 14.24, 25.82, 0.51, 2.54, 3.70, 5.17, 7.28, 9.74, 14.76, 26.31, 0.81, 2.62, 3.82, 5.32, 7.32, 10.06, 14.77, 32.15, 2.64, 3.88, 5.32, 7.39, 10.34, 14.83, 34.26, 0.90, 2.69, 4.18, 5.34, 7.59, 10.66, 15.96, 36.66, 1.05, 2.69, 4.23, 5.41, 7.62, 10.75, 16.62, 43.01, 1.19, 2.75, 4.26, 5.41, 7.63, 17.12, 46.12, 1.26, 2.83, 4.33, 5.49, 7.66, 11.25, 17.14, 79.05, 1.35, 2.87, 5.62, 7.87, 11.64, 17.36, 1.40, 3.02, 4.34, 5.71, 7.93, 11.79, 18.10, 1.46, 4.40, 5.85, 8.26, 11.98, 19.13, 1.76, 3.25, 4.50, 6.25, 8.37, 12.02, 2.02, 3.31, 4.51, 6.54, 8.53, 12.03, 20.28, 2.02, 3.36, 6.76, 12.07, 21.73, 2.07, 3.36, 6.93, 8.65, 12.63, 22.69.

**Figure 7:**
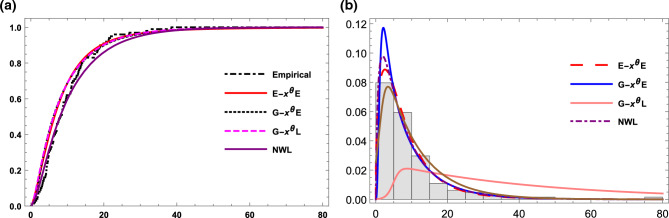
(**a**) The empirical and the estimated cdf for data set IV. (**b**) The histogram and estimated pdf for data set IV.

Plots of the fitted cdf’s and pdf’s for this data set are shown in Fig. 7. The calculated parameter values and goodness of fit measures are shown in Table 13. This table shows that, E-X$^{\theta }$E, G-X$^{\theta }$E, G-X$^{\theta }$L distributions are Fitted and perform well when examining this data set with a p-value greater than 0.05. While the NW-L distribution isn’t fitted to this data. However, the optimal model to acquire the best assessment of the data is the E-X$^{\theta }$E model, which has the smallest values of -ll, AIC, BIC, AICc and K-S statistics, as well as the highest p-value of all the examined models. Figure 7 supports the results in Table 13

**Table 13:** Parameter estimate and goodness-of fit statistics data set IV.

Model	Estimates	-${\hat{ll}}$	AIC	BIC	AICc	K-S	p-value
E-X$^{\theta }$E$(\alpha , \theta )$	(0.0602, 0.41)	410.0	824.05	824.1	824.4	0.037	0.999
G-X$^{\theta }$E$({\alpha }, \beta , {\theta })$	(2.745, 1.238, 0.605)	420.5	847.05	845.56	847.98	0.036	0.994
G-X$^{\theta }$L$({\alpha }, \beta , {\theta },c)$	(1.216, 0.28, 0.585, 0.046)	411.4	830.8	827.59	832.4	0.047	0.926
NW-L(${\alpha },{\beta }$)	(0.10613, 3.199)	414.1	836.28	832.37	832.7	0.129	0.026

### Using discrete data

Applications to count data sets from different fields are examined to demonstrate the practical applicability of the proposed models presented in Table 8. The R software is used to acquire the statistical results. MLE’s, Akaike Information Criteria (AIC), Bayesian Information Criteria(BIC), $\chi ^{2}$ test with associated p-values are performed to compare the flexibility of distributions. For competitive considerations, the data were also fitted to the Gamma distribution and some other discrete competitive distribution shown in Table 14.

**Table 14:** The discrete competitive distributions.

Model	Abbreviation	By
Discrete extended Erlang-truncated Exponential	DEETE	^19^
Discrete inverted Nadarajah–Haghighi (DINH) Distribution	INH	^20^
Discrete extended odd Weibull Exponential	DEOWE	^21^
Discrete binomial Exponential II	DBiExII	^22^
Discrete Poisson–Mirra distribution	PMiD	^23^

### Data set V

This data set represents the survival times of 44 patients suffering from head and neck cancer who retreated using a combination of radiotherapy. This data is taken from Ref.^24^. The data are:

12 32 37 24 24 74 81 26 41 58 63 68 78 47 55 84 155 159 92 94 110 127 130 133 140 112 119 146 173 179 194 195 339 432 209 249 281 319 469 725 817 519 633 1776

**Figure 8:**
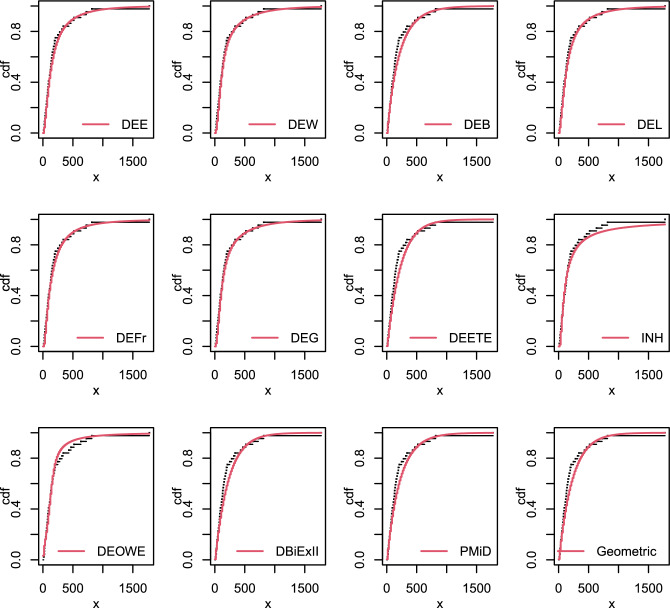
The empirical cdf’s of some fitted distributions for data set V.

Figure 8 illustrates the empirical and estimated cdf of this data. Table 15 displays the calculated parameters as well as the goodness-of-fit values. From the outcomes of this Table, we conclude that the DEE, DEW, DEB, DEL, DEFr, DEG, DEETE, INH, DEOWE, DBiEXII, PMiD and Geometric distributions are Fitted and perform quite well for evaluating this data. The DEE distribution is selected as the best model for the data because it has the smallest values of the goodness-of-fit statistics and the highest p-value among all the competitive distributions.

**Table 15:** Parameter estimate and goodness-of fit statistics data set V.

Model	Estimates	-$\varvec{{\hat{ll}}}$	AIC	BIC	df	$\varvec{\chi ^{2}}$	p-value
DEE($\alpha , \theta , \beta$)	(0.0382, 0.658, 0.0107)	277.25	561.44	566.79	3	0.989	0.804
DEW($\alpha , \theta , \beta , \eta$)	(0.0467, 0.623, 0.002, 1.345)	277.25	562.49	569.64	2	1.037	0.595
DEB($\alpha , \theta , \beta , \eta$)	(0.102, 0.764, 0.012, 2.733)	280.93	569.86	576.99	2	4.003	0.135
DEL($\alpha , \theta , \beta , \eta$)	(0.0428, 0.641, 80.58, 0.0001)	277.74	563.47	570.61	2	0.981	0.613
DEFr($\alpha , \theta , \beta , \eta$)	(0.299, 0.3999, 0.642, 140.49)	277.31	562.61	569.75	2	1.298	0.523
DEG($\alpha , \theta , \beta , \eta$)	(0.047, 0.622, 1.585, 0.0175)	277.25	562.50	569.64	2	1.099	0.577
DEETE($\alpha , \beta , \lambda$)	(1.078, 1.1685, 0.0040)	282.03	570.06	575.41	3	4.506	0.212
INH($\alpha , \beta$)	(1.0229, 76.911),	279.46	562.92	566.49	4	2.268	0.687
DEOWE($\alpha , \theta , \beta$)	(334.34, 0.0415, 0.380)	282.04	570.08	575.43	3	3.47	0.324
DBiExII($\alpha , \theta$)	(0.0044, 0.00085)	282.09	568.19	571.76	4	4.739	0.315
PMiD($\alpha , \theta$)	(1.7 $\times 10^{-6}$, 0.005)	281.68	567.37	570.93	4	4.497	0.343
Geometric(*p*)	(0.0044)	282.09	566.19	567.97	5	4.741	0.448

### Data set VI

These data show the time intervals in days between coal mine explosions from March 15, 1851 to March 22, 1962, inclusive. This time range is 40,550 days long. So the data consists of 190 numbers totaling 40,549. The 0 occurs because two incidents occurred on June 6, 1875. These findings are presented in Ref.^25^. The data set is; 0 1 1 2 2 3 4 4 4 6 7 10 11 12 12 12 13 15 15 16 16 16 17 17 18 19 19 19 20 20 22 23 24 25 27 28 29 29 29 31 31 32 34 34 36 36 37 40 41 41 42 43 45 47 48 49 50 53 54 54 55 56 59 59 61 61 65 66 66 70 72 75 78 78 78 80 80 81 88 91 92 93 93 95 95 96 96 97 99 101 108 110 112 113 114 120 120 123 123 124 124 125 127 129 131 134 137 139 143 144 145 151 154 156 176 182 186 187 188 189 190 193 194 197 202 203 208 215 216 217 217 217 218 224 225 228 232 233 250 255 275 275 275 276 286 292 307 307 312 312 315 324 326 326 329 330 336 345 348 354 361 364 368 378 388 420 431 456 462 467 498 517 536 538 566 632 644 745 806 826 871 952 1205 1312 1358 1630 1643 2366

**Figure 9:**
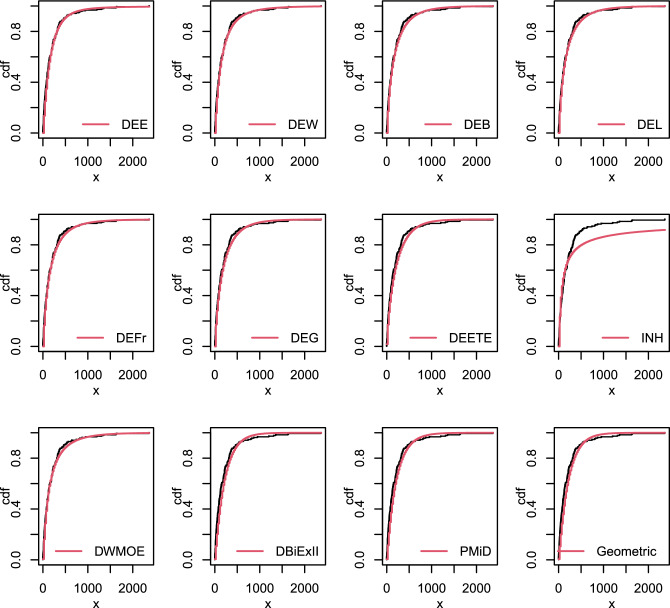
The empirical cdf’s of some fitted distributions for data set VI.

The summary statistics for this data is shown in Table 16. Figure 9 depicts the fitted cdf plots of the proposed models compared with the DEETE, INH, DEOWE, DBiEXII, PMiD and Geometric distributions for the data set VI. The estimated parameter values and the goodness of fit measures are presented in Table 16. According to the results in this table, the DEE, DEW, DEB, DEL, DEFr, DEG and DEOWE distributions are fitted to the data. The DEE distribution is the best option among other competitive models as it has the greatest p-value, while the DEG has the smallest other goodness of fit statistics, as shown by the findings in Table 16.

**Table 16:** Parameter estimate and goodness-of fit statistics for data set VI.

Model	Estimates	-$\varvec{\hat{ll}}$	AIC	BIC	df	$\varvec{\chi ^{2}}$	p-value
DEE($\alpha , \theta , \beta$)	(5.56, 1.1 $\times$ e−9, 0.0011)	1187.28	2380.55	2390.26	10	14.35	0.158
DEW($\alpha , \theta , \beta , \eta$)	( 0.121, 0.5, 0.062, 0.53)	1185.55	2379.09	2392.04	9	13.84	0.128
DEB($\alpha , \theta , \beta , \eta$)	(0.206, 0.607, 0.041, 1.072)	1186.28	2380.57	2393.51	9	13.84	0.128
DEFr($\alpha , \theta , \beta , \eta$)	(0.273, 0.498, 0.22, 521.3)	1185.92	2379.84	2392.78	9	13.66	0.135
DEL($\alpha , \theta , \beta , \eta$)	(0.60, 0.549, 0.024, 0.359)	1186.17	2380.33	2393.28	9	13.59	0.137
DEG($\alpha , \theta , \beta , \eta$)	(0.015, 0.799, 6.29, 3.009)	1181.77	2371.54	2384.46	9	14.12	0.118
DEETE($\alpha , \beta , \lambda$)	(0.72, 0.51, 0.007)	1191.46	2388.91	2398.62	10	18.54	0.047
INH($\alpha , \beta$)	(0.616,44.56)	1224.12	2452.25	2458.72	11	64.07	0
DEOWE($\alpha , \theta , \beta$)	(0.204, 0.0017, 1.065)	1185.99	2377.98	2387.69	10	15.63	0.111
DBiExII($\alpha , \theta$)	(0.005,0.0003)	1197.83	2399.67	2406.14	11	23.62	0.014
PMiD($\alpha , \theta$)	(2e−6, 5.3 $\times$ e03)	1195.17	2394.34	2400.82	11	20.91	0.034
Geometric(*p*)	(0.0046)	1197.83	2397.67	2400.9	12	23.63	0.023

### Data set VII

This data derived from a study performed in the lab on male mice who were given a 300 roentgen radiation exposure and were 5–6 weeks old. This information describes additional causes of death than the two primary causes: Thymic lymphoma and reticulum cell sarcoma. This data were examined by Ref.^26^. The data are:

40 42 51 62 163 179 206 222 228 252 249 282 324 333 341 366 385 407 420 431 441 461 462 482 517 517 524 564 567 586 619 620 621 622 647 651 686 761 763

**Figure 10:**
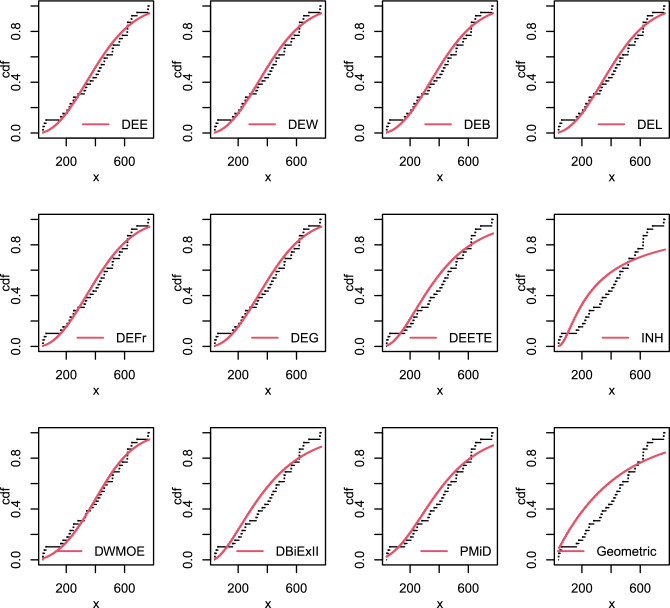
The empirical cdf’s of some fitted distributions for data set VII.

Figure 10 shows the fitted cdf plots of the suggested models compared with the DEETE, INH, DWMOE, DBiEXII, PMiD and Geometric distributions to this data set. Table 17, shows the estimated values of parameters as well as the goodness of fit statistics.

This table shows that, with the exception of the INH and Geometric distributions, all distributions are fitted and performed well when examining these data with p-values greater than 0.05. However, The PMiD distribution is the best option among other competitive models as it has the greatest p-value, as shown by the findings in Table 17.

**Table 17:** Parameter estimates and goodness-of fit statistics for data set VII.

Model	Estimates	-$\varvec{\hat{ll}}$	AIC	BIC	df	$\varvec{\chi ^{2}}$	p-value
DEE($\alpha , \theta , \beta$)	(8.98, 1.073, 3.3 $\times 10^{-7}$)	263.12	532.34	537.33	3	1.469	0.689
DEW($\alpha , \theta , \beta , \eta$)	(0.082, 1.84, 3.6 $\times 10^{-5}$, 0.23)	263.17	534.34	540.99	2	1.469	0.479
DEB($\alpha , \theta , \beta , \eta$)	(3 $\times 10^{-6}$, 2.075, 2.017, 1.08)	263.17	534.34	540.99	2	1.475	0.478
DEL($\alpha , \theta , \beta , \eta$)	(1.25, 1.07, 0.962, 2.4 $\times 10^{-6}$)	263.18	534.35	541.00	2	1.484	0.476
DEFr($\alpha , \theta , \beta , \eta$)	(3 $\times$ e−6, 2.07, 1.498, 0.015)	263.17	534.34	540.99	2	1.472	0.479
DEG($\alpha , \theta , \beta , \eta$)	(0.01, 1.44, 0.63, 2.2 $\times 10^{-6}$)	263.17	534.34	540.99	2	1.478	0.478
DEETE($\alpha , \beta , \lambda$)	(2.57,1.207,0.003)	267.13	540.25	545.24	3	2.383	0.497
INH($\alpha , \beta$)	(1.037, 218.32)	281.98	567.97	571.29	4	17.65	0.001
DWMOE($\alpha , \theta , \beta$)	(0.005,1.0293)	266.79	537.59	540.91	4	2.86	0.582
DBiExII($\alpha , \theta$)	(0.005, 1.0293)	266.79	537.59	540.91	4	2.859	0.582
PMiD($\alpha , \theta$)	(0.0004, 0.0067)	264.96	533.92	537.35	4	1.592	0.810
Geometric(*p*)	(0.0024	273.93	549.86	551.52	5	13.01	0.023

### Data set VIII

These data are the yields from 70 consecutive runs of a batch chemical process (see^25^). The data are 23 23 25 34 35 35 36 37 38 38 38 39 40 40 41 41 43 44 44 45 45 45 45 45 48 48 49 50 50 50 50 50 50 51 51 51 51 51 52 53 54 54 54 54 55 55 55 55 56 57 58 58 59 59 59 59 60 60 62 64 64 64 68 71 71 71 74 74 80.

**Figure 11:**
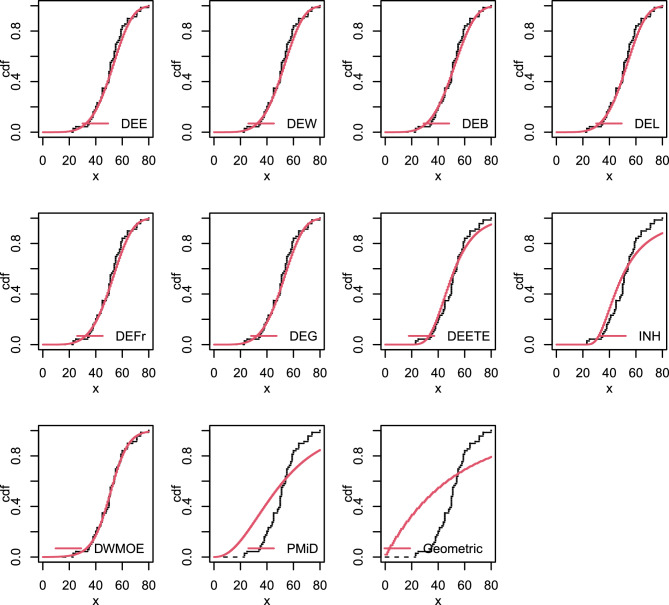
The empirical cdf’s of some fitted distributions for data set VIII.

The empirical and estimated cdf for the this data is shown in Fig. 11. The estimated parameters and goodness-of-fit measurements are also included in Table 18.

This Table shows that, the DEE, DEW, DEB, DEL, DEFr, DEG, DEETE, and DEOWE distributions are Fitted and perform quite well for evaluating this data with p-values larger than 0.05. The DEOWE distribution is selected as the best model for these data because it has the smallest -ll, AIC, BIC, and $\chi 2$ values and the highest p-value.

**Table 18:** Parameter estimates and goodness-of fit statistics for data set VIII.

Model	Estimates	-$\varvec{\hat{ll}}$	AIC	BIC	df	$\varvec{\chi ^{2}}$	p-value
DEE($\alpha , \theta , \beta$)	(0.025, 3.794, 1.6 $\times 10^{-7}$)	268.8	543.59	550.29	5	8.078	0.152
DEW($\alpha , \theta , \beta , \eta$)	(0.723, 4.10, 5.7 $\times 10^{-9}$, 0.69)	268.8	545.59	554.53	4	8.075	0.089
DEB($\alpha , \theta , \beta , \eta$)	( 2 $\times 10^{-8}$, 4.42, 3.11, 0.071)	269.2	546.31	555.24	4	8.280	0.082
DEL($\alpha , \theta , \beta , \eta$)	(4.18, 3.79, 1.26, 7 $\times 10^{-10}$)	268.8	545.59	554.53	4	8.076	0.089
DEFr($\alpha , \theta , \beta , \eta$)	(1.6 $\times 10^{-8}$, 4.69, 0.011, 2.31)	268.8	545.64	554.58	4	8.023	0.091
DEG($\alpha , \theta , \beta , \eta$)	(5 $\times$ e−09, 4.73, 1.36, 1.157)	268.8	545.61	554.55	4	8.035	0.090
DEETE($\alpha , \beta , \lambda$)	(43.64, 6.971, 0.012)	274.4	554.89	561.59	5	10.01	0.075
INH($\alpha , \beta$)	(3.363, 43.62)	286.2	576.43	580.90	6	24.36	0.0004
DEOWE($\alpha , \theta , \beta$)	(683.3, 0.128, 1.156)	268.3	542.63	549.34	5	5.910	0.315
PMiD($\alpha , \theta$)	(220.3, 0.059)	304.3	612.66	617.13	6	70.99	0
Geometric(*p*)	(0.019)	340.8	683.51	685.74	7	173.9	0

## Summary and conclusion

In this research, a new way for generating distributions is developed with high degree of flexibility that would be very useful in modeling real data in various fields. The new family of distributions is called the T-X$^{\theta }$ family suggested with extra shape parameters $\theta$ for bounded and positive unbounded random variables. Several specific sub-families and sub-models of the proposed family are presented including the Exponential-X$^{\theta }$ Exponential distribution which was selected and studied in details. The parameters of this distribution were estimated using the MLE method. A simulation study was also conducted to investigate the efficiency as well as behavior of estimates. The discretized T-X$^{\theta }$ family of distributions has been proposed and some discrete models of the family were defined. As an example, the discrete Exponential Exponential, DEE, distribution, a three-parameter discrete distribution derived, various alternative graphs of the DEE’s pmf and hazard functions were shown. Eight different actual data sets were used to demonstrate the effectiveness of some members of the suggested continuous and discrete members of the family vs some other distributions. In general, the results indicate that the proposed distributions are highly flexible, provide accurate results and can fit various types of data. The new models achieved a closer match for all data sets.

## Data Availability

The datasets analyzed in this study were a re-analysis of existing data, which are are included in Refs.^24–26^ and available at locations cited in the reference section.
